# Colon Cancer Metastatic to the Thyroid Gland in the Setting of Pathologically Diagnosed Papillary Thyroid Cancer: A Review and Report of a Case

**DOI:** 10.7759/cureus.7314

**Published:** 2020-03-18

**Authors:** Imran Zafar, Francis Buzad, Edward Weir

**Affiliations:** 1 Surgery, Texas A&M College of Medicine, Austin, USA; 2 Surgery, Saint David's North Austin Medical Center, Austin, USA; 3 Pathology, Clinical Pathology Associates, Austin, USA

**Keywords:** thyroid, colon, cancer, metastasis, malignancy, neoplastic, papillary, immunohistochemical staining, pulmonary metastasis, fine-needle aspiration

## Abstract

Colon cancer metastases to the thyroid gland are a particularly rare occurrence. Despite the relative amenability of the gland to clinical, radiologic, and pathologic assessment, preoperative distinction between primary and secondary thyroid neoplastic processes remains difficult. Here we describe a case of a patient with a known history of stage IV colon cancer with multiple pulmonary metastases, presenting with a thyroid lesion initially diagnosed as papillary thyroid cancer on fine-needle aspiration biopsy but found to be metastatic colonic adenocarcinoma on post-thyroidectomy pathologic evaluation utilizing immunohistochemical techniques. A review of the literature is also included.

## Introduction

Colorectal cancer (CRC) is the fourth most common cancer diagnosed in the United States, with an estimated incidence of 145,600 new cases in 2019 comprising 8.3% of all new cancer cases in the United States [1]. Although screening modalities and guidelines have improved in recent years, approximately 21% of patients with newly diagnosed CRC have distant metastatic disease at the time of diagnosis, with the most common sites of distal metastasis being the liver followed by the lungs [1].

Thyroid metastasis of all malignant tumors is a particularly rare occurrence, and thyroid metastasis of CRC specifically is even more so. This rarity is further complicated by the disparity between rates of autopsy-proven and clinically detectable thyroid metastases. Autopsy studies of malignant tumor cases have reported the frequency of thyroid metastasis to be between 1.9% and 9.5% [2,3]. However, evidence of thyroid metastases from non-thyroid malignancies is reported to be significantly rarer in clinical practice, ranging from 1.4% to 3% of all detected thyroid neoplasms [4]. In accordance with this, a cytologic study consisting of 25,000 fine-needle aspirations performed on thyroid nodules identified an overall incidence of 0.1% to be metastatic neoplastic disease [5]. As a consequence of the rarity of this phenomenon, the literature regarding thyroid metastasis of CRC deals nearly exclusively with case reports.

## Case presentation

In July 2014, a 57-year-old Caucasian woman with a family history of metastatic CRC in a younger brother underwent a screening colonoscopy that revealed adenocarcinoma of the right ascending colon. Preoperative computed tomography (CT) at the time showed enlarged lymph nodes in the right colonic mesentery and a 6.3-mm solitary pulmonary nodule in the right lower lobe, without evidence of clear distant metastatic disease. She underwent a robotic-assisted laparoscopic right hemicolectomy in August 2014. Pathologic examination revealed a 3.5-cm grade 2 tumor invading through the muscularis propria into the pericolonic adipose tissue and a positive mesenteric margin with four tumor deposits. There was lymphovascular and perineural invasion noted, with involvement of four to sixteen lymph nodes. There was an additional incidental finding of an 0.55-cm well-differentiated neuroendocrine (carcinoid) tumor of the appendix. She was pathologically staged with pT3, pN2a, pMX stage IIIB adenocarcinoma of the right ascending colon, which was found to be KRAS mutation-positive with microsatellite instability, and pT1a appendiceal well-differentiated stage IA neuroendocrine carcinoma.

Further staging workup with positron emission tomography-CT (PET-CT) in September 2014 revealed a persistent solitary pulmonary nodule in the right lower lobe that did not characterize metabolically. Adjuvant chemotherapy with fluorouracil, leucovorin, and oxaliplatin (FOLFOX) was started, and a restaging PET-CT in March 2015, after completion of 10 cycles of adjuvant therapy, showed complete resolution of the previously seen right lower lobe pulmonary nodule. Of note, there was symmetrical increased metabolic activity in the thyroid gland reported at this time, suggesting possible underlying multinodular goiter.

Restaging PET-CT in September 2015 revealed a metabolically active and enlarging pulmonary nodule in the right lower lobe consistent with metastatic disease, and subsequent biopsy confirmed metastatic colonic adenocarcinoma. At this time, she was reclassified as stage IV disease, KRAS-positive with microsatellite instability. The patient underwent a wedge resection of the right lower lobe of the lung and excision of a station 9 right lymph node in November 2015, with subsequent pathologic examination confirming metastatic colonic adenocarcinoma and a solitary benign lymph node.

Restaging CT scans from June 2016 through August 2017 showed the appearance and subsequent enlargement of two subcentimeter right and left upper lobe pulmonary nodules, with PET-CT confirming metabolic activity in bilateral apical lung fields and in a level III left neck lymph node. Of note, a diffusely enlarged thyroid with diffuse increased PET activity was noted on this scan, and maintained to be suggestive of multinodular goiter. The patient completed a four-day regimen of stereotactic body radiation therapy, and adjuvant therapy with fluorouracil, leucovorin, irinotecan, and bevacizumab (FOLFIRI) was started, with 10 cycles completed by March 2018.

**Figure 1:**
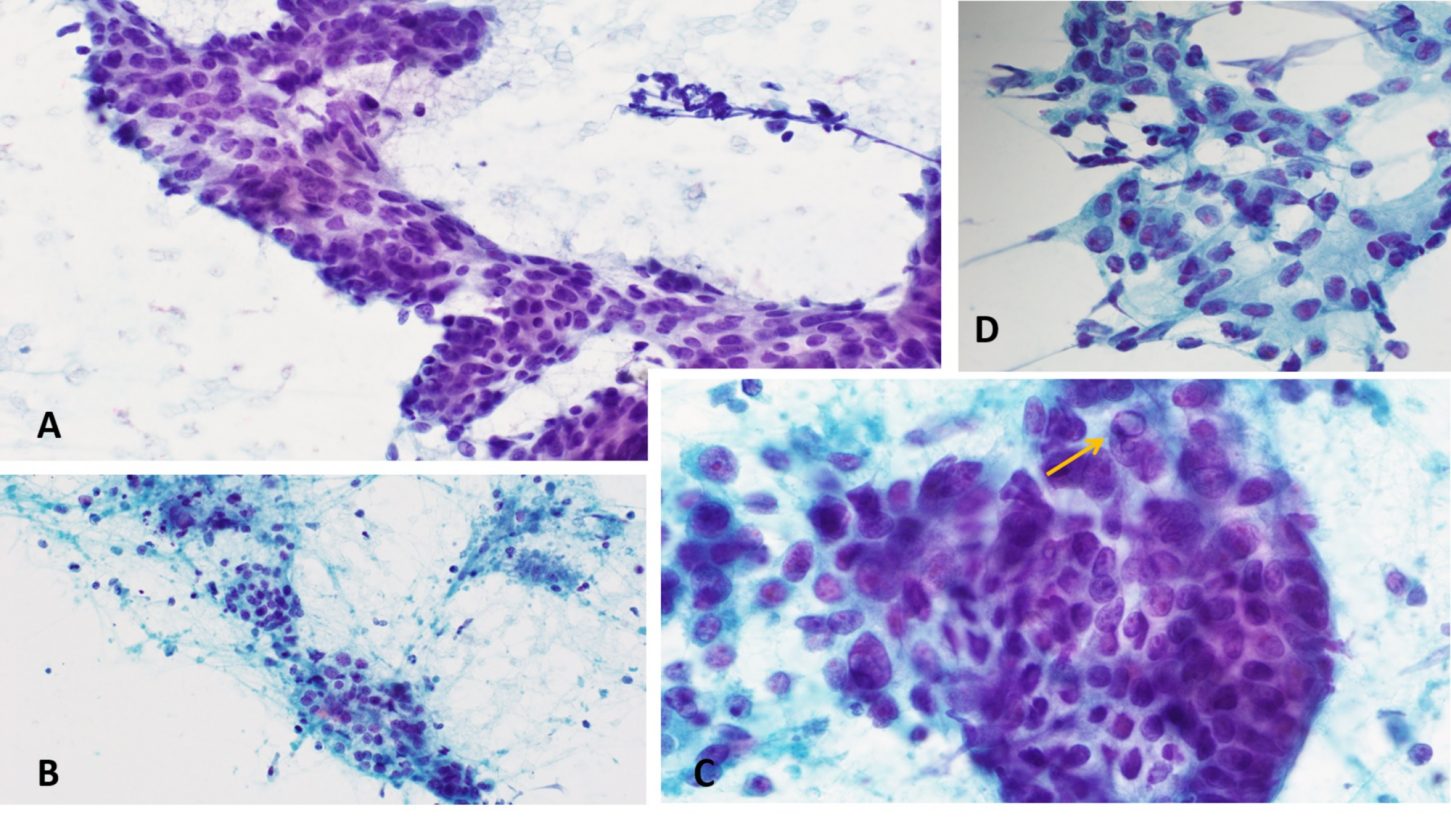
Fine-needle aspiration biopsy of thyroid tissue (A) Cytologic preparations of a thyroid fine-needle aspiration show colorectal adenocarcinoma cells in three-dimensional clusters, some of which demonstrate branching architecture that resembles papillae in papillary thyroid carcinoma. (B) A background of necrotic debris surrounds many cell clusters. (C, D) Though high-power examination reveals scattered cells with clearing of the nuclear cytoplasm, typical of papillary thyroid carcinoma (C, arrow), the striking nuclear pleomorphism is unusual for papillary carcinoma. Papanicolaou stains magnification 40X and 100X.

In July 2019, the patient presented with complaint of a neck mass and difficulty swallowing. She underwent fine-needle aspiration of the thyroid isthmus and a left lobe nodule identified on ultrasound, with both tissue samples pathologically identified as papillary thyroid cancer (Figure 1).

**Figure 2:**
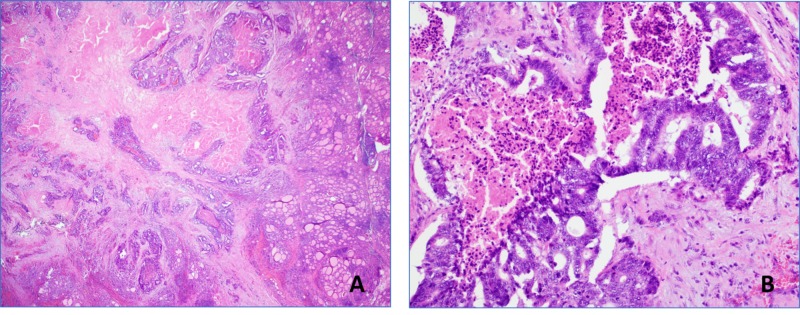
Colorectal adenocarcinoma primary tumor and thyroid metastasis (A) Colorectal adenocarcinoma metastatic to the thyroid gland. Thyroid parenchyma is present on the right side of the field (hematoxylin & eosin, magnification 2X). (B) Colorectal adenocarcinoma with intraluminal necrosis (hematoxylin & eosin, magnification 10X).

The patient underwent a total thyroidectomy in August 2019, and pathologic analysis of the gross specimen confirmed two foci of metastatic colonic adenocarcinoma at the posterior surgical margin with a background of chronic lymphocytic thyroiditis (Figure 2). The foci were determined to be 4.3 and 1.5 cm in size, and found to involve one of two right paratracheal lymph nodes.

**Figure 3:**
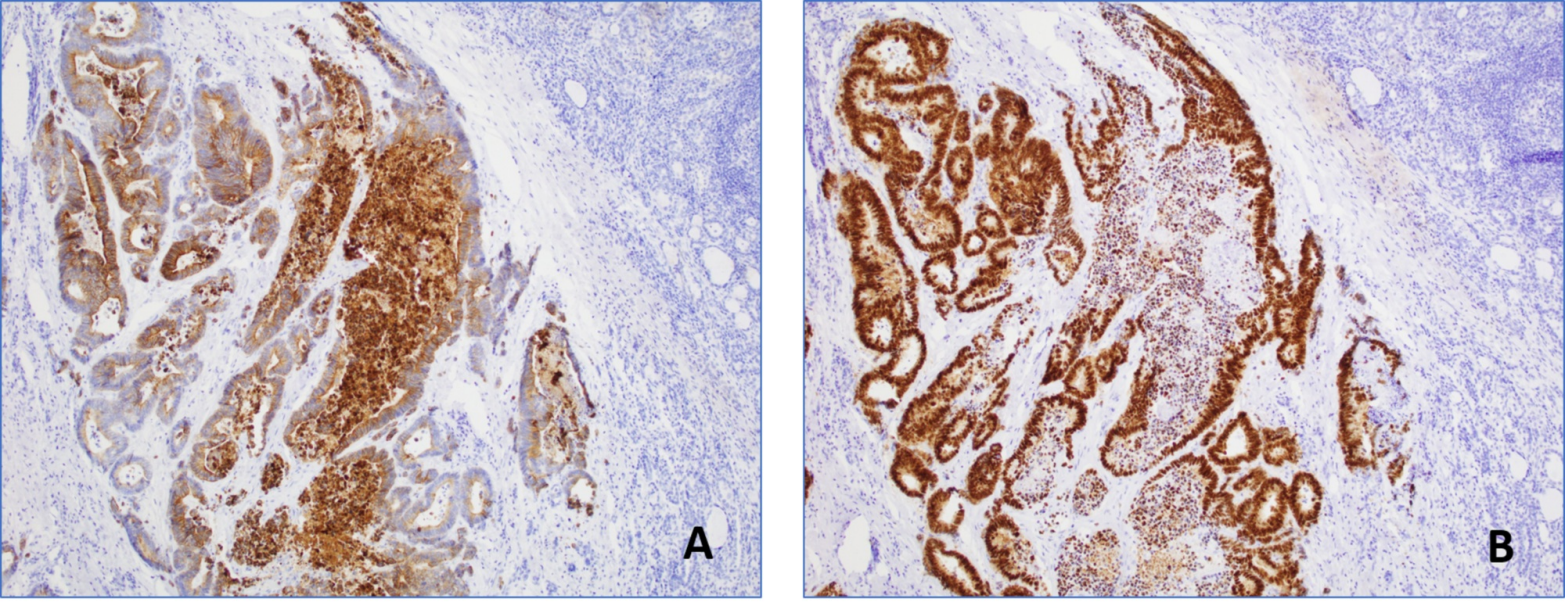
Immunohistochemical stains of thyroid tissue Immunohistochemical expression of cytokeratin 20 (A, magnification 10X) and CDX-2 (B, magnification 10X) confirming colorectal origin.

Neoplastic origin was confirmed with immunohistochemical staining techniques, with the cells staining positive for cytokeratin 20 (CK20) and CDX-2, and negative for cytokeratin-7 (CK7), transcription termination factor 1 (TTF-1), and paired box gene 8 (PAX-8), consistent with a primary colonic malignancy (Figure 3).

## Discussion

Two possible explanations for the rarity of thyroid metastasis of malignant tumors have been proposed. The mechanical explanation reasons that although the abundant vascularization of the thyroid gland allows the gland to come in contact with distal neoplastic cells, the blood flow rate of 560 ml/100 g tissue/min across the gland prevents neoplastic cells from remaining fixed within it [2,6]. The chemical explanation proposes that the high oxygen saturation and high iodine content of the gland creates an immunoprotective environment, precluding the propagation of neoplastic cells [2]. These hypotheses may serve to partially reconcile the disparity between the rates of clinical and autopsy diagnoses of thyroid metastasis presented in the literature mentioned above.

A retrospective analysis of 21 detailed cases of thyroid metastasis from CRC conducted by Keranmu et al. provides insight into certain patterns and associations of this neoplastic process. The median age of the patients at the time of diagnosis of thyroid metastasis was 59 years [7]. In this study, 71.4% of patients had a primary tumor distributed throughout the distal colon or rectum. Furthermore, 81.0% of patients showed concomitant pulmonary metastasis and in 76.2% of these patients, the thyroid metastasis was clinically detected after the lung metastasis. Of note, a literature review of 31 cases of CRC metastatic to the thyroid by Kumamoto et al. report the most common site of origin, excluding the rectum, as the right ascending colon [8]. Overall, the patient and tumor metrics surrounding the discovery and location of the colonic primary and associated metastatic lesions presented by these studies are in accordance with our own case.

Considering the mechanical and chemical hypotheses put forth by Willis et al., in conjunction with the link between pulmonary and thyroid metastases of CRC described by Keranmu et al., provides a novel insight into the route of spread for this neoplastic process [2,7]. The most common distal metastatic location of CRC, excluding lymph nodes, is the liver followed by the lungs. Although the route of metastasis to the liver is believed to be hematogenous through the portal venous circulation, the route of metastasis to the thyroid remains uncertain [9]. Given that clinical data suggest that thyroid metastasis is discovered after a known pulmonary metastasis, it is conceivable that the portal circulation allows the primary lesion to seed to the liver, from where it can disseminate systemically to the lungs and finally the thyroid. However, there have been reported cases of thyroid metastasis of CRC without any other clinically detectable metastases, including examples of thyroid metastasis of CRC occurring before clinically detectable lung metastasis [7]. Given these findings, the vertebral venous system has been proposed as a medium for metastatic spread - a route fully independent of entrance to the thoracic or abdominal cavities that completely bypasses the portal vein, pulmonary vein, and vena cava [9].

In addition to a mechanical basis for thyroid metastasis of CRC, there may also exist a genetic component. Prior studies have demonstrated a significantly higher degree of KRAS mutations in primary tumors of patients with lung metastases, with 57% of metastatic cases exhibiting KRAS mutations, compared to 37% of cases exhibiting the wild-type gene [10]. Given the findings by Keranmu et al. suggesting that thyroid metastasis occurs after pulmonary metastasis, it is conceivable that mutation-induced constitutive KRAS activity plays a genetic role in the aggressive metastasis of CRC to the thyroid gland. This proposed hypothesis warrants further research. 

Due to the relative ease of evaluation of the thyroid gland through physical exam, ultrasound-mediated imaging, and fine-needle aspiration biopsy, multiple modalities are used to monitor and diagnose any aberrancies arising in this gland. Nonetheless, preoperative distinction between primary ands secondary thyroid neoplastic processes remains difficult to differentiate using cytology alone, as in our presented case, and thyroidectomy is often performed for definitive diagnosis. Initial stages of diagnosis with results from ultrasound and fine-needle aspiration can be misleading, as evident in our case. In situations such as these, there may be indication for additional tests such as biomarker-specific immunohistochemical staining, assuming adequate cellularity of the sample. Staining for thyroid-specific immunomarkers including TTF-1, CK7, thyroglobulin (Tg), and PAX-8 can be helpful in confirming a primary thyroid malignancy [11]. Similarly, immunohistochemical staining positive for markers including CK20 and homeobox protein CDX-2 points to a metastatic colonic malignancy [11].

Thyroid metastasis is considered to be a late event, diagnosed years after the diagnosis of the initial primary disease and is often detected with synchronous or metachronous metastasis in other organs [12]. The primary treatment of solitary thyroid metastases of non-thyroid malignancies is surgical, either through partial or total thyroidectomy, and aims to balance tumor burden with survival given the poor prognosis. A report of a large case series from the Mayo Clinic concluded that, of patients with thyroid metastases, those who underwent thyroid resection had better outcomes compared with those who underwent more conservative therapies [13]. Treatment of metastatic thyroid lesions involves balancing the effects of the local tumor burden often presenting as clinical events with the known rates of survivorship given the progression and prognosis of the disease. In our case as with many others, thyroidectomy was a valid choice of treatment.

## Conclusions

Although the thyroid gland is susceptible to a multitude of benign pathologies, neoplastic processes should never be overlooked in the context of concerning history, physical exam, or diagnostic study findings. Most commonly, neoplastic processes are those of primary thyroid origin. Although rare occurrences, documented cases of metastatic cancer to the thyroid do exist in the literature, and accurate detection is key. A subset of these cases involves metastatic colonic adenocarcinoma, which should be considered in any patient with a history of a primary colonic malignancy, particularly if there is a concurrent history of pulmonary metastasis. In such scenarios, care should be taken to utilize thyroid tissue biopsy methods to yield samples with adequate cellularity and retention of microscopic architecture. Subsequent pathologic evaluation may be supplemented with immunohistochemical techniques to uncover molecular specificities that are crucial in differentiating between primary and secondary neoplastic processes.
